# High Glucose Promotes the Ferroptosis and Dysfunction of Endothelial Cells by Downregulating SLC3A2 and Promoting the Development of Nephropathy

**DOI:** 10.1155/ije/1186113

**Published:** 2025-07-16

**Authors:** Yingying Ji, Qi Wang, Jun Wang, Qi Zhang, Ying Jiang, Peipei Luan

**Affiliations:** Wuxi Central Rehabilitation Hospital, The Affiliated Mental Health Center of Jiangnan University, Wuxi 213151, Jiangsu, China

**Keywords:** diabetic nephropathy, endothelial dysfunction, ferroptosis, high glucose, single-cell RNA sequencing

## Abstract

**Background:** Diabetic nephropathy, a leading cause of end-stage renal disease, is a major health concern. Its early-stage signs are unclear. Endothelial dysfunction, an early indicator, is suitable for early detection and intervention. However, current treatments mainly focus on glycemic and blood pressure control, lacking specific methods for targeting this dysfunction.

**Method:** We reanalyzed GSE13535 dataset, which has single-cell RNA-seq of high-glucose-exposed endothelial cells. HUVECs were cultured in high-glucose and TNF-α. We conducted RNA extraction, qPCR, western blotting, iron measurement, TUNEL assay, and bioinformatics analysis. An antiferroptosis drug was used in STZ-treated diabetic mice.

**Results:** Single-cell RNA-seq showed early endothelial cell dysfunction, along with ENDMT, cytokine release, and ferroptosis activation. SLC3A2 was identified as a key; its reduced expression was linked to more inflammation, fibrosis, and ferroptosis. Diabetic mice had low SLC3A2 and more ferroptosis in glomerular endothelial cells. A STAT1 inhibitor alleviated endothelial dysfunction.

**Conclusion:** Endothelial dysfunction and ferroptosis are crucial in diabetic nephropathy. Identifying SLC3A2 as a key regulator gives insights into potential treatments. Fludarabine shows promise. Further research on mechanisms and strategies is needed to improve patient outcomes.

## 1. Introduction

Diabetes mellitus is a serious metabolic disease, which is characterized by high glucose resulting from absolute or relative lack of insulin. The population with diabetic mellitus was predicted to be approximately 439 million around the world by 2030 [1, 2]. We all know that prolonged diabetes could cause damage to microvascular complications, which mainly include diabetic nephropathy (DN). DN served as the most complication of diabetic mellitus, which was the leading cause of final stage of renal disease around the world. Actually, many papers illustrated that early detection and treatment of DN were crucial for the patients who were sensible to develop DN for their better control [3]. Although the early stage of nephropathy is decisive for the DN progress, but the effective detection or targeting marker of early-stage DN was still unclear [4–6]. Some laboratory tests, like serum creatinine and urine protein, are not obvious enough to subtly detect early renal manifestations of diabetes [7]. Except the laboratory tests, the histological signs of DN including glomerular basement membrane (GBM) thickening, mesangial dilation, and loss of progenitor cells, these could also serve as a histology diagnosed criterion [8, 9]. But no matter the laboratory tests or histological signs, these were still not the early stages of DN detection's diagnose criterion.

The glomerular filtration barrier is mainly composed of three layers: the endothelial layer, the GBM, and podocytes. Among them, podocytes and endothelial cells (ECs) cross-talk through molecular signals to maintain the filtration function of the glomerular filtration barrier [10, 11]. High glucose affects the three-layer structure of the glomerular filtration barrier, as well as the cross-talk of podocyte and ECs, so one of the early events of glomerular destruction in DN is EC dysfunction [12]. However, the strategy for DN is mainly to control blood glucose and lower blood pressure, and there is no treatment for endothelial dysfunction in the early stage, so it is of great significance to detect EC dysfunction in the early stage of DN so that we can diagnose and intervene early [13]. However, data on early stage of EC dysfunction in glomerular filtration have not been fully explored.

Recent studies highlight ferroptosis, an iron-dependent form of regulated cell death driven by lipid peroxidation, as a critical contributor to renal injury in chronic kidney diseases, including DN. Ferroptosis is characterized by glutathione depletion, GPX4 inactivation, and iron overload, processes exacerbated by high glucose and inflammation. However, the molecular mechanisms linking ferroptosis to glomerular endothelial cell (GEC) dysfunction in early DN remain poorly understood. SLC3A2, a subunit of the cystine/glutamate antiporter system X_c_^−^, is essential for maintaining intracellular glutathione levels and redox balance. Downregulation of SLC3A2 has been implicated in ferroptosis in cancer and neurodegenerative diseases, yet its role in diabetic endothelial injury is unexplored [14, 15].

In this study, we aimed to use scRNA-seq to identify markers for early detection and treatment during the process of EC dysfunction. We further conducted relevant experiments both in vitro and in vivo to explore the effects of these screened marker genes or pathways on the glomerular filtration barrier in DN.

## 2. Methods

### 2.1. Cell Culture

Human umbilical vein endothelial cells (HUVECs) from ScienCell (Cat#8000) were cultured in ScienCell's endothelial cell medium (ECM) (Cat#1001), supplemented with fetal bovine serum (FBS) (Cat#0025), endothelial cell growth supplement (ECGS) (Cat#1052), and penicillin/streptomycin (P/S) (Cat#0503). The cells were maintained in a humidified incubator at 37 °C with 5% CO_2_. To investigate the effects of elevated glucose and TNF-α, HUVECs were exposed to complete ECM supplemented with 25 mM glucose (Sigma, Cat#G8644) and 10 ng/mL of TNF-α (Perprotech, Cat#300-01A). For the STAT1 inhibitor experiment, the STAT1 inhibitor fludarabine (MCE, Cat#HY-B0069) was utilized after the cells were exposed to high glucose and TNF-α. Specifically, we referenced the work of Sophia et al. [16] who systematically evaluated fludarabine effects in cell cultures, showing that concentrations of 5–20 μM induced measurable cellular responses while maintaining viability. Thus, the cells were treated with fludarabine (5 μM) for 24 h. For overexpression of SLC3A2, we generated a plasmid carrying SLC3A2. Finally, the cells were harvested for RNA or protein extraction for further analysis. In this study, the human SLC3A2 cDNA (NCBI accession: NM_001012661.2) was cloned into the pcDNA3.1(+) vector (Invitrogen) using EcoRI and XhoI restriction sites, followed by Sanger sequencing verification (Sangon Biotech) to ensure plasmid integrity. For transfection, HUVECs at 70%–80% confluence were transfected with the SLC3A2-pcDNA3.1(+) plasmid or empty vector (control) using Lipofectamine 3000 (Invitrogen) in Opti-MEM, adhering to the manufacturer's protocol. Transfection efficiency was validated 48 h post-transfection via qRT-PCR and Western blot, confirming successful SLC3A2 overexpression.

### 2.2. RNA Extraction and qPCR Analysis

Total RNA was extracted using TRIzol reagent (Cat#15596026, Thermo Fisher Scientific) as per the manufacturer's instructions. Subsequently, 1 μg of total RNA underwent reverse transcription using HiScript III RT SuperMix (Cat#R323, Vazyme). The mRNA expression levels were normalized to GAPDH and were calculated using 2^(−ΔΔCt)^ method. Quantitative PCR was carried out utilizing ChamQ Universal SYBR qPCR Master Mix (Cat#Q711, Vazyme), with primer details available in Supporting Table 1.

### 2.3. Western Blotting

The cells underwent lysis using RIPA buffer (P0013C, Beyotime). Following lysis, 10 μg of total proteins were separated on a polyacrylamide gel and subsequently transferred to PVDF membranes (ThermoFisher Scientific). These membranes were then blocked with 5% bovine serum albumin in PBS for 1 h at room temperature. Next, they were incubated overnight at 4°C with primary antibodies. Subsequently, the membranes were washed and further incubated with HRP-conjugated secondary antibodies for 1 h at room temperature. Western blot densities were quantified using ImageJ software (v1.52a, National Institutes of Health) and normalized to housekeeping proteins.

### 2.4. Iron Measurement

The measurement of iron levels was performed using the Phen Green SK (PGSK) fluorescent probe (Cat#P14313, Thermo Fisher) for intracellular analysis. For flow cytometry analysis, cells were initially harvested and rinsed with PBS following a 15 min incubation with 2 μM PGSK. Subsequently, samples were assessed using the BD FACSAria III. For fluorescent imaging, cells were fixed in 4% paraformaldehyde (PFA) for 15 min after the incubation with 2 μM PGSK, followed by three washes with PBS prior to observation.

### 2.5. TUNEL Assay

Cell apoptosis analysis in transfected cells was conducted utilizing the TUNEL assay (Cat#116848179, Roche). Post-transfection, cells were fixed in 4% paraformaldehyde at 4°C for 15 min. The TUNEL staining was performed using the Roche TUNEL kit. TUNEL-positive cells were enumerated using a fluorescence microscope (Olympus).

### 2.6. Bioinformatics Analysis

The datasets were analyzed using Seurat v3.1.1 in R [17]. We excluded cells with fewer than 200 expressed genes or with mitochondrial genes accounting for over 10% of total expressed genes. Additionally, we addressed potential doublets, including those resulting from encapsulation or incomplete dissociation, using the DoubletFinder package (version 2.0.2) [18]. Filters were applied to cells in each dataset, including the number of genes, the number of UMIs, and the percentage of reads mapping to mitochondrial genes. Raw UMI counts per cell were normalized by total counts, multiplied by 10,000, and then natural log was transformed with a pseudo-count of 1 added. Highly variable genes were identified based on mean expression and dispersion criteria. Genes with mean expression between 0.05 and 10 and dispersion between 1.5 and 20 were designated as highly variable. Batch effect removal across samples was performed using integration analysis implemented in Seurat v3.1.1. Integration anchors were identified based on the union of highly variable genes across datasets, with a maximum of 1500 genes used in total, and the first 20 dimensions from canonical correlation analysis. Integration was carried out using the identified anchors. Two-dimensional UMAP was employed for visualization using the first 20 principal components derived from the integrated assay. Graph-based clustering was performed on the integrated dataset. In scRNA-seq analysis, a shared nearest neighbor (SNN) graph was constructed with 50 nearest neighbors and 20 dimensions of principal components as input. Clusters were identified using the graph with a resolution parameter of 0.4. Differential gene expression between clusters was assessed using the MAST test implemented in Seurat, with each gene required to be expressed in at least 25% of cells in either group. Genes with |log2foldchange|  >  1.5 were considered significantly differentially expressed, with Bonferroni corrected *p* value  <  0.05 indicating statistical significance. Mitochondrial genes (prefix “MT-”) were excluded.

### 2.7. Animals

The experimental procedures followed the National Institutes of Health guidelines for the Care and Use of Laboratory Animals [19]. Eight-week-old C57BL/6J wild-type (WT) mice received intraperitoneal injections of streptozotocin (STZ, 75 mg/kg) for 5 consecutive days. After 20 weeks, when fasting blood glucose levels exceeded 16 mmol/L, indicating diabetes, the mice were euthanized, and kidney and body weights were measured. Urinary albumin levels and serum biochemical parameters were assessed using a colorimetric enzymatic assay system at the study's conclusion. For the fludarabine in vivo injection experiments, mice received subcutaneous injections of 0.1 mg/g for 10 consecutive days following STZ injection.

### 2.8. Morphology, Immunohistochemistry, and Immunofluorescence Staining

For morphological analysis of the kidneys, they were fixed with a 4% paraformaldehyde solution and subsequently embedded in paraffin for sectioning. Sections were then stained with hematoxylin and eosin (HE) or periodic acid Schiff (PAS). Imaging of the stained sections was conducted to detect histological changes in kidney tissues. Additionally, Masson staining was performed to evaluate matrix deposition in the interstitial areas. For immunofluorescence staining, sections underwent antigen retrieval and were incubated overnight at 4°C with primary antibodies. The following day, they were incubated with secondary antibodies for 1 h at room temperature. Nuclei were labeled with DAPI from Vector Laboratories, and images were visualized using a confocal microscope (Nikon). A list of all antibodies used is provided in Supporting Table 2.

### 2.9. Fasting Glucose Test

Following a 12 h fasting period, blood samples were collected from the caudal artery of the mice using a needle. Glucose levels in the blood samples were assessed using a glucose meter.

### 2.10. Statistical Analysis

The experimental data were presented as mean ± SD. All statistical analyses were performed using GraphPad Prism software (La Jolla, USA). The statistical test, p value, and sample size are stated in the figure legends.

## 3. Results

### 3.1. Single-Cell RNA Sequencing Identified Dysfunctional ECs With Activated Ferroptosis Signaling Pathway

**Figure 1:**
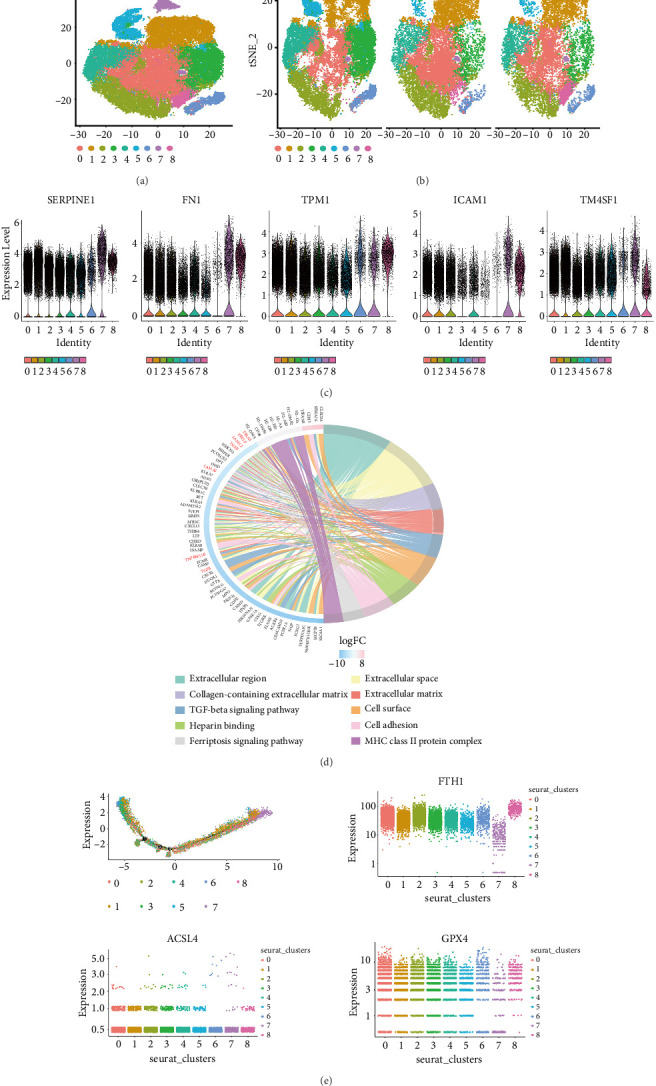
Single-cell RNA sequencing data screen one unique group of cells highly expressing inflammatory and stem-cell markers. (a) Uniform manifold approximation and projection (UMAP) visualization of all single-cell RNA sequencing (scRNA-seq) data from endothelial cells. (b) The UMAP results are split by different days. (c) The violin plot reveals the expression of different markers. (d) The enriched pathway and DEGs extracted from Group 7. (e) The pseudotime analysis of different genes' expressions in the process of TNF + high glucose stimulated.

We analyzed a GEO dataset (GSE13535) containing scRNA-seq data of different stages (0, 3, and 7 days after stimulation) of HUVECs exposed to high glucose and inflammatory environment. These stimulations mimic the diabetic environment inducing dysfunction of ECs in glomerular filtration barrier. We found that after exposing to TNF and high glucose stimulation, the HUVECs could be divided into nine groups of cells (Figure 1(a)). After the HUVECs challenged with high glucose and TNF stimulation at different time points. The results showed the cell clusters change as the time goes on (Figure 1(b)). As the time goes by, we found that the number of group 7 cells gradually increased after challenge. We found that group 7 cells highly expressed ENDMT markers of Serpine1, Fn1, and Tpm1. We also found that the group 7 cells could highly express inflammatory markers, such as Icam1 and proliferated genes, such as Tm4sf (Figure 1(c)). We found that the Group 7's Fn1 expression was higher significantly than group 8 (Supporting Figure 1A). Many studies identified this group of cells as dysfunction ECs in DN glomerular filtration barrier. Meanwhile, we found that the group 4 cells expressed CD31, and the number of ECs was decreasing gradually within 7 days, which is considered as healthy ECs (Supporting Figure 1B). We extracted the characteristic genes of the group 7 cells to perform gene enrichment analysis and displayed them using circle diagrams. We noticed one interesting signaling pathway: the ferroptosis signaling pathway was highly upregulated in group 7 cells (Figure 1(d)). We also observed that the highly expressed genes in groups 7 cells were mainly enriched in signaling pathways such as inflammation or fibrosis, such as the TGF-beta signaling pathway and extracellular matrix generation. We also conducted the pseudo-time analysis; we found that genes related to ferroptosis, such as GPX4 and FTH1 were both downregulated significantly in group 7 cells, while ACSL4 was highly regulated significantly in group 7 (Figure 1(e)). These data all indicated that the ferroptosis signaling pathway was activated in the early dysfunction stage of ECs and might influence the progress of diabetic DN (Figure 1(e)).

### 3.2. SLC3A2 Is a Potentially Valuable Gene That Contributes to the Phenotype Dysfunction and Ferroptosis of ECs

**Figure 2:**
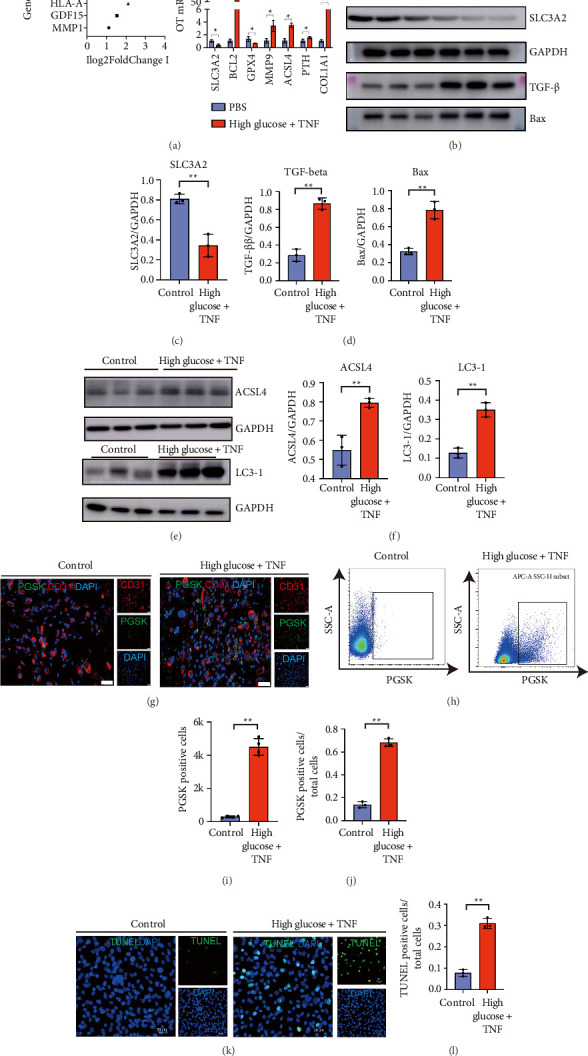
SLC3A2 is a potentially valuable gene that influences the phenotype of Group 7. (a) Left, the list of DEGs ranked by the absolute value of log2FoldChange; right, qPCR analysis of HUVECs treated with PBS or high glucose + TNF. *n* = 3/group. (b, e) Western blot analysis of HUVECs treated with PBS or high glucose + TNF. *n* = 3/group. (c, d) Quantification of (b). (f) Quantification of (e). (g) Fluorescence staining of iron measurement on HUVECs treated with PBS or high glucose + TNF. Green, PGSK; red, CD31; blue, DAPI. *n* = 3/group. (h) Flow cytometry analysis of iron measurement on HUVECs treated with PBS or high glucose + TNF. *n* = 3/group. (i) Quantification of (g). (j) Quantification of (h). (k) TUNEL analysis on HUVECs treated with PBS or high glucose + TNF. Green, TUNEL; blue, DAPI. *n* = 3/group. (l) Quantification of K. All data are mean ± SD. (a, c, d, f, i, j, l) Two-tailed unpaired Student's *t*-test. ^∗^*p* < 0.05 and ^∗∗^*p* < 0.01.

Because the group 7 ECs could be considered as dysfunctional ECs, and the group 4ECs could be identified as normal functional ECs, we screened the DEGs by comparing the genes between both groups of cells. The DEG-screened criterion was log_2_FC > 1.5, and adjusted *p* value < 0.05. Excluding the mitochondria-related genes starting with MT, the most promising one was SLC3A2 (the lowest *p* value and the largest change in LogFC value) (Figure 2(a)). The high glucose and TNF were used to challenge the HUVECs in vitro to mimic the microenvironment of GECs in DN environment. After challenge, we found that the challenged HUVECs highly expressed genes related to inflammation (MMP9) and apoptosis-related genes (BCL2) and also highly expressed fibroblast-related genes (Col1a1). These data all show that after stimulation, ECs underwent significant phenotype changes of proinflammation, fibrosis, and increased apoptosis. At the same time, we noticed that there are also significant changes in genes related to ferroptosis-related proteins such as SLC3A2, GPX4, and ACSL4 (Figure 2(a)). At the same time, after the cells challenged with high glucose combined with TNF, the protein expression level of TGF-β and the apoptotic protein level of Bax were significantly increased. Meanwhile, we found that ferroptosis marker of SLC3A2 was significantly decreased. The ferroptosis-related proteins ACSL4 and LC3-1 were also significantly upregulated. These results indicated that HUVECs under diabetic condition challenge exhibited dysfunction phenotype including significant ferroptosis, inflammatory, and ENDMT (Figures 2(b), 2(c), 2(d), 2(e), 2(f)). Furthermore, the PGSK green fluorescent probes were used to detect the iron ion content between both groups of control and high glucose condition challenged through flow cytometry and confocal microscopy. We found that after HUVECs were challenged with high glucose and TNF condition, the iron ion content of cells was increased significantly (Figures 2(g), 2(h), 2(i), 2(j)). At the same time, we found that the apoptotic rate of HUVECs was also increased significantly after challenge. These data showed that the TNF combined with high glucose stimulation could cause the ferroptosis signaling pathway activated in HUVECs, accompanied by a significant decreased expression of Slc3a2. Some reference papers identified the downregulation of Slc3a2 as a key marker causing ferroptosis [20–22]. Therefore, we concluded that HUVECs underwent ferroptosis and was transdifferentiated to dysfunction phenotype under diabetic challenge of high glucose and inflammatory cytokine (Figures 2(k), 2(l)). So, the comparative transcriptomics and functional assays revealed that diabetic conditions (high glucose/TNF-α) induce endothelial dysfunction via SLC3A2 suppression, triggering ferroptosis, inflammation, and fibrosis—key hallmarks of early DN. These findings prompted further investigation into Slc3a2's therapeutic potential in vivo.

### 3.3. Activated Ferroptosis Signaling Pathway in Glomerular ECs, With Lower Expression of Slc3a2 in Diabetic Mice

**Figure 3:**
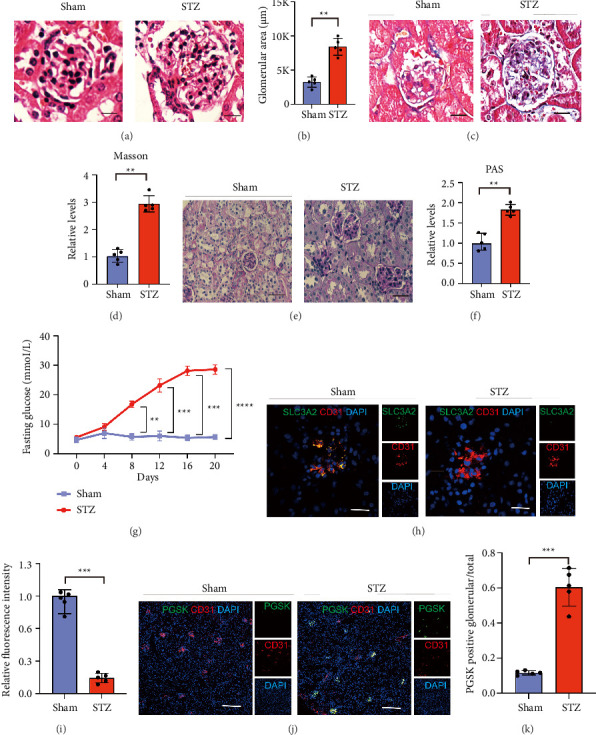
Increased ferroptosis in endothelial cells is accompanied by a low expression of SLC3A2 in STZ-treated diabetic mice. H&E (a), Masson (c), and PAS (e) staining of mice kidney. *n* = 5/group. (b) Quantification of (a). (d) Quantification of (c). (f) Quantification of (e). (g) Fasting blood glucose of WT-Mock and WT-STZ mice during the 20-week period, *n* = 5/group. (h) Fluorescence staining of the kidney from WT-Mock and WT-STZ mice. Green, SLC3A2; red, CD31; blue, DAPI, *n* = 5/group. (i) Quantification of (h). (j) Fluorescence staining of iron measurement on kidney from WT-Mock and WT-STZ mice. Green, PGSK; red, CD31; blue, DAPI. *n* = 5/group. (k) Quantification of (j). Quantification of (k). All data are mean ± SD. (b, d, f, i, k) Two-tailed unpaired Student's *t*-test. (g) Two-way ANOVA with Bonferroni post hoc test. ^∗∗^*p* < 0.01 and ^∗∗∗^*p* < 0.001.

We used the STZ to induce high glucose in mice. After 20 weeks, we observed both upregulation of 24 h albuminuria and urea albumin/creatinine ratios in STZ-treated diabetic WT(WT-STZ) mice, compared with those in the control group (*n* = 5 mice/group, 12,623 vs.38652 mg/d, *p*=0.003; 38.4213 vs.23.4359 g/mol, *p*=0.008, respectively) (Supporting Figure 3A&B). Histologic analysis showed that glomerular volume was larger and Bowman's capsule was more compressed in diabetic mice compared with control mice (Figures 3(a), 3(b)). PAS and Masson staining showed that mesangial expansion and fibrosis area in diabetic mice were significantly higher than that in STZ-treated mice compared with the control group of mice (Figures 3(c), 3(d), 3(e), *n* = 5 mice/group). Compared with mice not injected with STZ, the blood glucose of mice injected with STZ increased more significantly during the same period (Figure 3(g), *n* = 5 mice/group). From these data, we could conclude that the STZ induced high glucose caused DN phenotype in diabetic mice.

At the same time, we found that ECs expressed lower expression of SLC3A2 in the kidney of DN mice, and at the same time, the iron ion content was relatively higher in GECs of DN mice (Figures 3(h), 3(i), 3(j), 3(k)). These all indicated that in the hyperglycemic environment of DN, GECs underwent significant ferroptosis, accompanied by a low expression of SLC3A2. So, we found that STZ-induced diabetic mice developed classical DN features including hyperglycemia, albuminuria, and glomerular pathology while exhibiting reduced SLC3A2 expression and elevated iron accumulation in GECs—demonstrating ferroptosis activation during DN progression.

### 3.4. Fludarabine Impeded Endothelial Dysfunction by Inhibiting Ferroptosis of GECs Through Regulating the STAT-RELA Signaling Pathway

**Figure 4:**
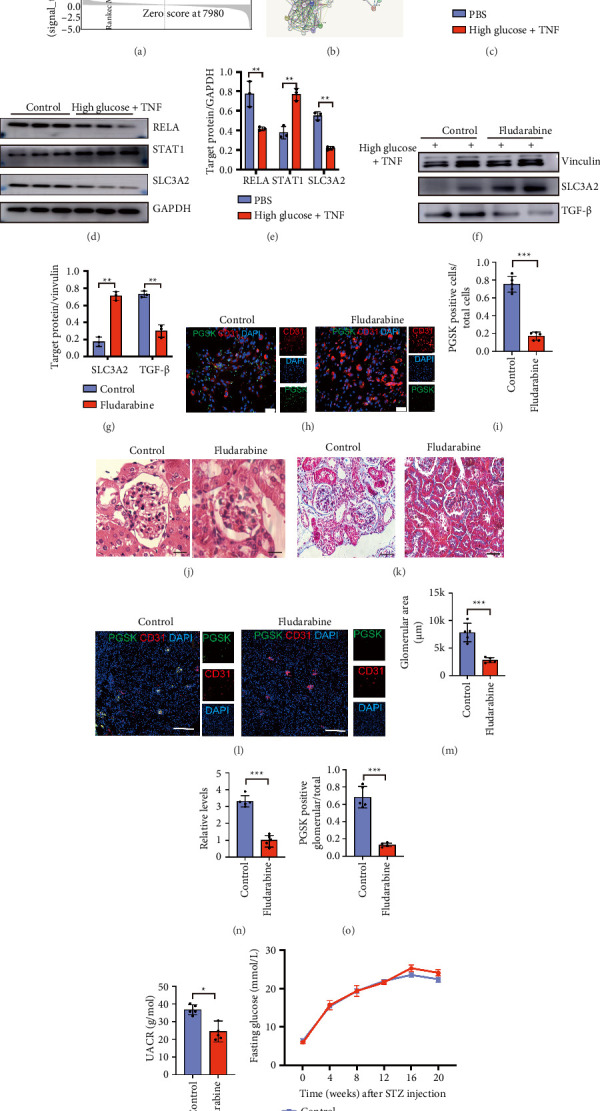
Fludarabine alleviates endothelial cell–mesenchymal transition caused by ferroptosis of endothelial cells by inhibiting the STAT signaling pathway and promoting the increase of RELA expression. (a) The GSEA analysis of enriched genes extracted from Group 7. (b) The predicted string results from web. (c) qPCR analysis of HUVECs treated with PBS or high glucose + TNF. *n* = 3/group. (d) Western blot analysis of HUVECs treated with PBS or high glucose + TNF. *n* = 3/group. (e) Quantification of (b). (f) Western blot analysis of HUVECs treated with or without fludarabine in the presence of high glucose + TNF. *n* = 3/group. (g) Quantification of (h). Fluorescence staining of iron measurement on HUVECs treated with or without fludarabine in the presence of high glucose + TNF. Green, PGSK; red, CD31; blue, DAPI. *n* = 3/group. (i) Quantification of (h). H&E (j) and Masson (k) staining of mice kidney, *n* = 5/group. (l) Fluorescence staining of iron measurement on the kidney from WT-STZ mice treated with or without fludarabine. Green, PGSK; red, CD31; blue, DAPI, *n* = 5/group. (m) Quantification of (j). (n) Quantification of (k). (o) Quantification of (l). (p) The urea albumin/creatinine ratio of WT-STZ mice treated with or without fludarabine. (q) The quantification of the plasma glucose level between two groups. All data are mean ± SD. (c, e, g, i, m, n, o, p) Two-tailed unpaired Student's *t*-test. ^∗^*p* < 0.05, ^∗∗^*p* < 0.01, and ^∗∗∗^*p* < 0.001.

We then used the GSEA enrichment analysis to screen the activated signal pathways between the group of normal ECs (group 4) and dysfunction ECs (group 7); we found that the gene profile in group 7 was mainly enriched in the JAK-STAT signaling pathway (Figure 4(a)). We then used the STRING website (STRING: functional protein association networks) to explore the potential regulated relationship between SLC3A2 and STAT signaling pathways. We found that high glucose and TNF expression promoted the activation of JAK-STAT and inhibit SLC3A2 expression by upregulating the expression of RELA. These could promote the EC ferroptosis and severe inflammatory response, leading to EC dysfunction (Figure 4(b)). We challenged ECs with TNF + high glucose in vitro and found that ECs were downregulated expressing RELA at the transcriptome or protein level. At the same time, Slc3a2 expression was also significantly downregulated, while STAT1 was significantly upregulated (Figures 4(c), 4(d), 4(e)). Then, we used STAT1 inhibitor fludarabine to rescue dysfunctional ECs challenged with TNF + high glucose in vitro. We found that after stimulation with fludarabine, the expression of SLC3A2 was significantly increased and the expression of fibrosis marker TGF-β was significantly decreased (Figures 4(f), 4(g)).

When we overexpressed the SLC3A2 within ECs challenged with TNF + high glucose, we found that the SLC3A2 overexpression could rescue the dysfunctional phenotype within ECs (Supporting Figure 2 A&B). We also found that the number of ferroptosis ECs was also decreased after fludarabine rescued the ECs in vitro (Figure 4(h)). These all illustrated that the activation of STAT1 under TNF stimulation plays an important role in alleviating EC dysfunction. Furthermore, we injected fludarabine into the STZ-induced diabetic mice to conduct rescue experiments in vivo. We found that after STAT1 inhibition, histochemical staining showed that the size of the glomeruli of the mice was significantly reduced, and the Masson staining showed that the fibrosis area was significantly decreased (Figures 4(j), 4(k), 4(m), 4(n)). At the same time, we found that the use of fludarabine did alleviate the ferroptosis of ECs caused by high glucose and TNF stimulation. The iron ion load of ECs was significantly relieved (Figures 4(l), 4(o)), and we also found that the urinary protein of diabetic mice rescued with fludarabine was significantly reduced, indicating that kidney function has been improved (Figure 4(p)). Meanwhile, we could not observe significant difference of glycemia in diabetic mice between fludarabine rescued group and control group (Figure 4(q)). We checked the SLC3A2, TGF-β, Bax, and BCL2 by using RT-qPCR in kidney tissue in vivo; we found that after intervention with fludarabine, the SLC3A2 was upregulated, and TGF-β was downregulated, Bax was downregulated and Bcl-2 was upregulated; we incorporated this figure in Supporting Figure 3C. We also found that when ECs were stimulated with different dosages of fludarabine, the expression of the SLC3A2 was also increased correspondingly, while the TGF-β was decreased (Supporting Figure 2C). These data concluded that the fludarabine could impede the dysfunctional GECs through regulating the STAT/RELA signaling pathway, and inhibiting the ferroptosis and could rescue the DN phenotype in diabetic mice.

## 4. Discussion

DN, a formidable microvascular complication, silently progresses toward renal failure [23, 24]. However, because the diagnostic biomarkers or therapy strategies at the early stages are not well understood, the DN became hard to control the disease. In this study, we analyzed the scRNA-seq data of HUVECs challenged with diabetic risk factors including high glucose and TNF-α inflammatory cytokines. Then, we generated the single-cell transcriptome atlas for different stages of HUVECs challenged with DN risk factors. Furthermore, we used in vitro and in vivo to prove the pathophysiologic role of the most significant gene Slc3a2. We demonstrated that the dysfunctional ECs (group 7) were induced under the DN risk factors with the upregulation of inflammatory markers, apoptotic rate, and fibrosis index. And this group of cells was regarded as the earliest discovered cells in the EC dysfunctional process. Then, by using the GSEA analysis, we found that the ferroptosis signaling pathway was activated in this dysfunctional early stage of ECs (group 7).

We found the participation of several ferroptosis genes' change during this process, including GPX4, FTH1, and ACSL4, with this process [25–27]. Strengthening these findings, evidence from STZ-treated diabetic mice in vivo studies reinforced the reduced expression of SLC3A2 accompanied by elevated ferroptosis including the highly upregulated apoptotic rate and high iron ion ratio in the glomeruli of DN mice. We also found that the JAK-STAT signaling pathway played a role in SLC3A2's expression and subsequent ferroptosis initiation. In high glucose and TNF context, STAT activation suppresses SLC3A2, thus activating EC ferroptosis signaling pathway. Fludarabine, an inhibitor of STAT1, exhibited a profound rescuing effect in vivo, significantly rescuing the dysfunctional GECs of DN mice by increasing the SLC3A2 expression, thus inhibiting the fibrosis rate, mitigating the inflammatory environment, and inactivating the ferroptosis signaling pathway.

GEC dysfunction is vital in the process of development of DN [28–30]. GEC from the first cellular barrier directly contacts with the cells or circulating risk factors. So, any changes or disturbances in these circulating factors could induce the GECs' dysfunction. Considering GECs' important role that directly influences the podocyte function and its role in causing damage to podocyte and glomeruli, the GECs' dysfunction could be regarded as one of early detectable biomarkers in the process of DN [31]. So, these pathophysiological characteristic features made GECs as valuable detection or therapy target for rescuing or detecting DN. However, the early promising detectable biomarker is not well established in the DN. In this study, we found that Slc3a2 could serve as a promising biomarker that could reflect the early stages of DN in diabetic mice. And we also figured out that the high glucose risk factors could activate the STAT signaling pathway; then, it would cause the downregulated expression of Slc3a2. We also highlighted the fludarabine's potential function to mitigate disease severity and improve kidney function. We found that deeper investigations into the mechanism of the ferroptosis pathway in the process of ECs' dysfunction and prospective therapeutic interventions in early stages of DN could significantly broaden our ways to develop specific treatment roles to treat this kind of disease.

Our findings significantly advance the current understanding of DN pathogenesis by elucidating the mechanistic link among endothelial ferroptosis, STAT/Slc3a2 signaling, and early glomerular dysfunction. Previous studies have established that endothelial injury contributes to DN progression [32–34], yet the precise molecular events triggering endothelial dysfunction, particularly in the early stages, remained poorly defined. While prior research implicated ferroptosis in renal tubular injury [35–37], its role in ECs under diabetic conditions had not been thoroughly investigated. Here, we provide the first single-cell transcriptomic atlas of GECs exposed to DN risk factors (high glucose and TNF-α), revealing that ferroptosis activation is a hallmark of the earliest dysfunctional endothelial subpopulation (group 7). This extends prior bulk RNA-seq studies that reported ferroptosis-related gene changes in DN by pinpointing the specific cell state and stage at which ferroptosis initiates.

Clinically, our findings surpass previous observational associations between endothelial dysfunction and DN [38, 39] by proposing Slc3a2 as a functional biomarker and fludarabine as a targeted therapy. While earlier studies suggested JAK-STAT inhibitors as potential DN treatments [40, 41], none established their efficacy in rescuing endothelial ferroptosis or linked STAT1 to Slc3a2. Our in vivo data show that fludarabine not only inhibits STAT1 but also restores Slc3a2 expression, mitigating fibrosis, inflammation, and ferroptosis—effects not previously achieved with broad-spectrum antioxidants or iron chelators [42, 43]. This highlights the superiority of targeting upstream regulators (STAT1/Slc3a2) over downstream ferroptosis mediators.

While our study establishes SLC3A2 downregulation and ferroptosis activation as pivotal in high glucose/TNFα-induced endothelial dysfunction, bioinformatics analysis revealed concurrent enrichment of JAK-STAT and TGF-β signaling in dysfunctional cells, suggesting multipathway cross-talk. The STAT1 inhibitor fludarabine's rescue of both SLC3A2 expression and fibrosis (Figure 4) implies SLC3A2-dependent and -independent mechanisms, such as STAT1-mediated direct regulation of inflammatory/fibrotic genes. High glucose may also drive dysfunction via oxidative stress or miR-mediated pathways not explored here. These complexities underscore the need for future studies to dissect a broader regulatory network, ensuring a more comprehensive understanding of DN pathogenesis.

In conclusion, we offered a compelling narrative elucidating the indispensable role of ferroptosis and endothelial dysfunction in DN through the STAT/Slc3a2 ferroptosis signaling pathway. By identifying potential therapeutic targets and demonstrating fludarabine's efficacy, it opens up a promising path for clinical interventions. Subsequent research endeavors and clinical trials might further substantiate these findings, revolutionizing the landscape of managing and treating this debilitating condition.

## Data Availability

The data that support the findings of this study are available from the corresponding author upon reasonable request.

## Supporting Information

Supporting Information 1 Supporting Table 1: The list of primer sequence.

Supporting Information 2 Supporting Table 2: The list of antibodies and dilution ratio.

Supporting Information 3 Supporting Figure 1A: The violin plot reveals different expressions of Fn1 between Group 7 and Group 8. Supporting Figure 1B: The violin plot reveals the expression of PECAM1(CD31) markers.

Supporting Information 4 Supporting Figure 2A: The representative images of WB to detect whether the SLC3A2 overexpression could rescue the phenotype of ECs challenged by high glucose and TNF. Supporting Figure 2B: Quantification of A. Supporting Figure 2C: The relative expression of SLC3A2 and TGF-β of ECs challenged with different doses of fludarabine. All data are mean ± SE. ^∗^*p* < 0.05, ^∗∗^*p* < 0.01, and ^∗∗∗^*p* < 0.001.

Supporting Information 5 Supporting Figure 3A: Relative expression changes of albuminuria in vivo. Supporting Figure 3B: The relative expression change of creatinine/urea nitrogen ratio (mg/mmol) in vivo. Supporting Figure 3C: The relative expression of SLC3A2 and TGF-β Bcl-2, and Bax in the kidney tissue in vivo between the control group and fludarabine injected group. All data are mean ± SE. ^∗^*p* < 0.05, ^∗∗^*p* < 0.01, and ^∗∗∗^*p* < 0.001.
