# Supplementary figures and images for: High Glucose Promotes the Ferroptosis and Dysfunction of Endothelial Cells by Downregulating SLC3A2 and Promoting the Development of Nephropathy
Source: Int J Endocrinol. 2025 Jul 16;2025:1186113. doi: 10.1155/ije/1186113 (PMC12286665; doi:10.1155/ije/1186113)

Supplementary Figure 1

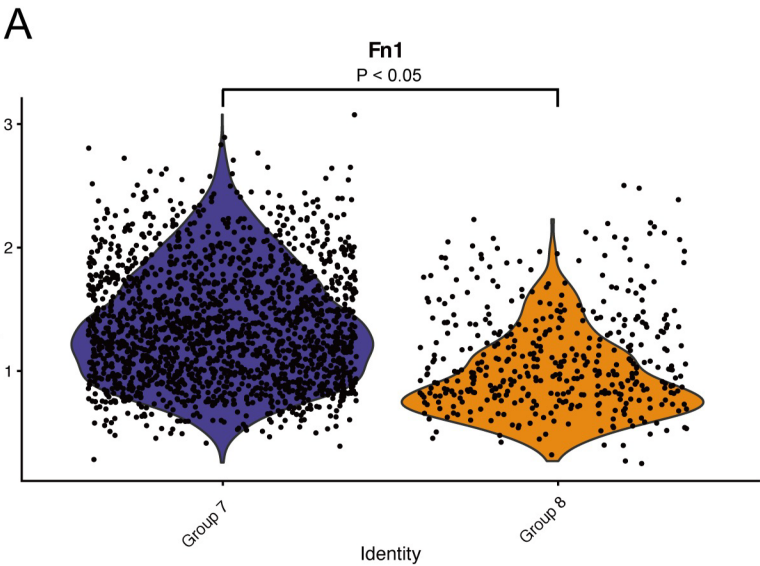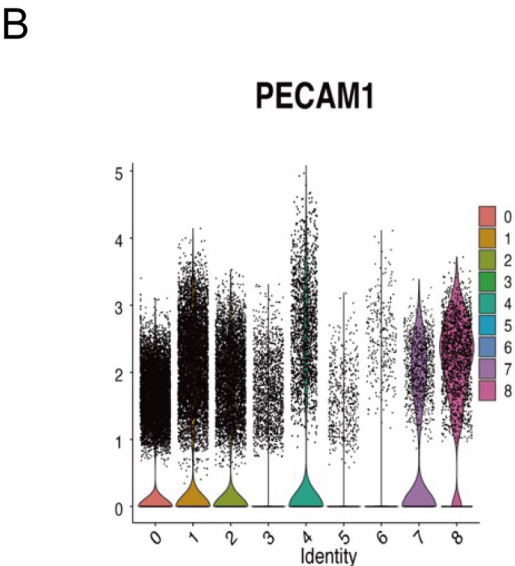

Supplement: Supporting Information 3 — Supporting Figure 1A: The violin plot reveals different expressions of Fn1 between Group 7 and Group 8. Supporting Figure 1B: The violin plot reveals the expression of PECAM1(CD31) markers. [file 1186113.f3.pdf]

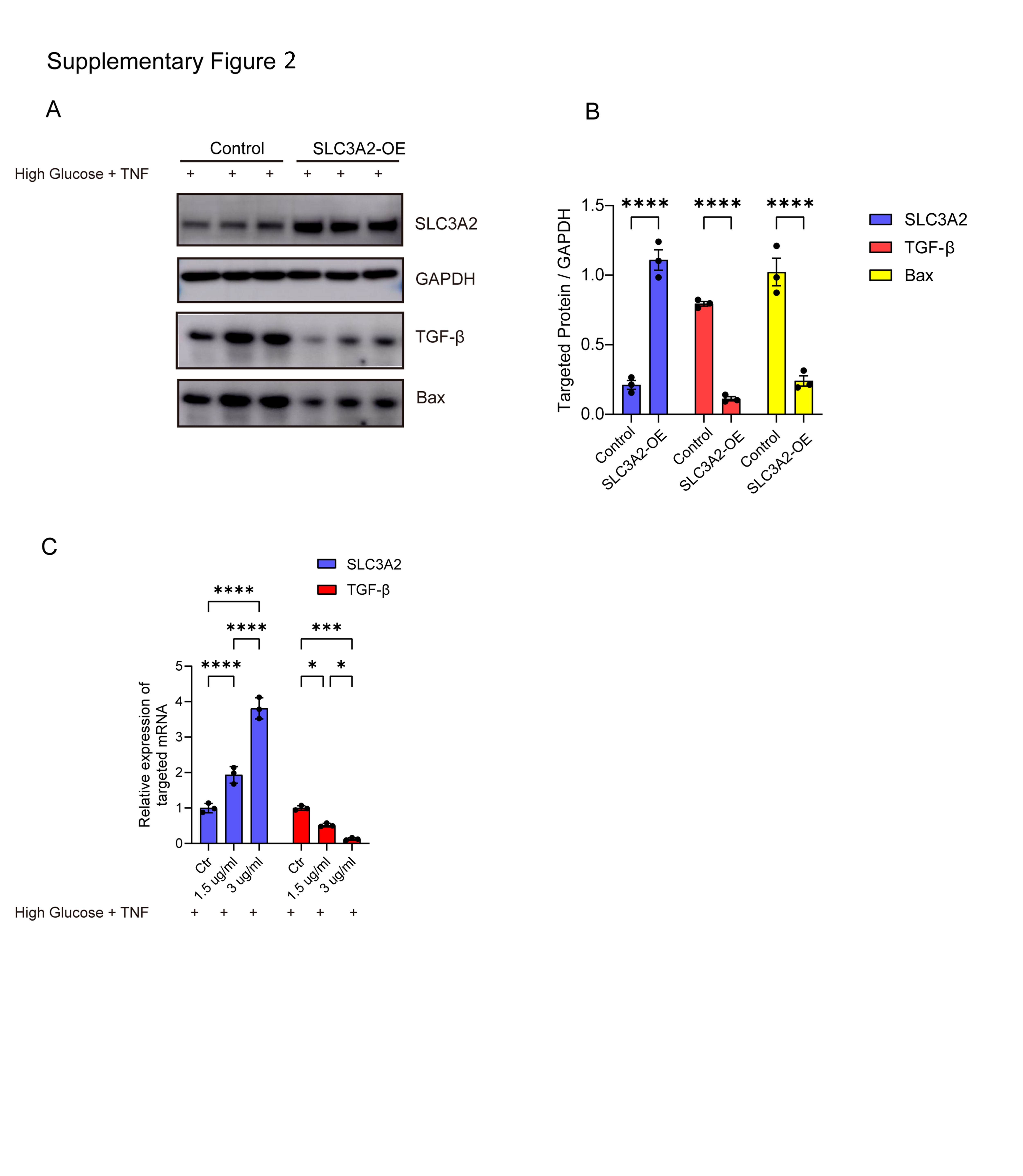

Supplement: Supporting Information 4 — Supporting Figure 2A: The representative images of WB to detect whether the SLC3A2 overexpression could rescue the phenotype of ECs challenged by high glucose and TNF. Supporting Figure 2B: Quantification of A. Supporting Figure 2C: The relative expression of SLC3A2 and TGF-β of ECs challenged with different doses of fludarabine. All data are mean ± SE. ∗p < 0.05, ∗∗p < 0.01, and ∗∗∗p < 0.001. [file 1186113.f4.png]

Supplementary Figure 3

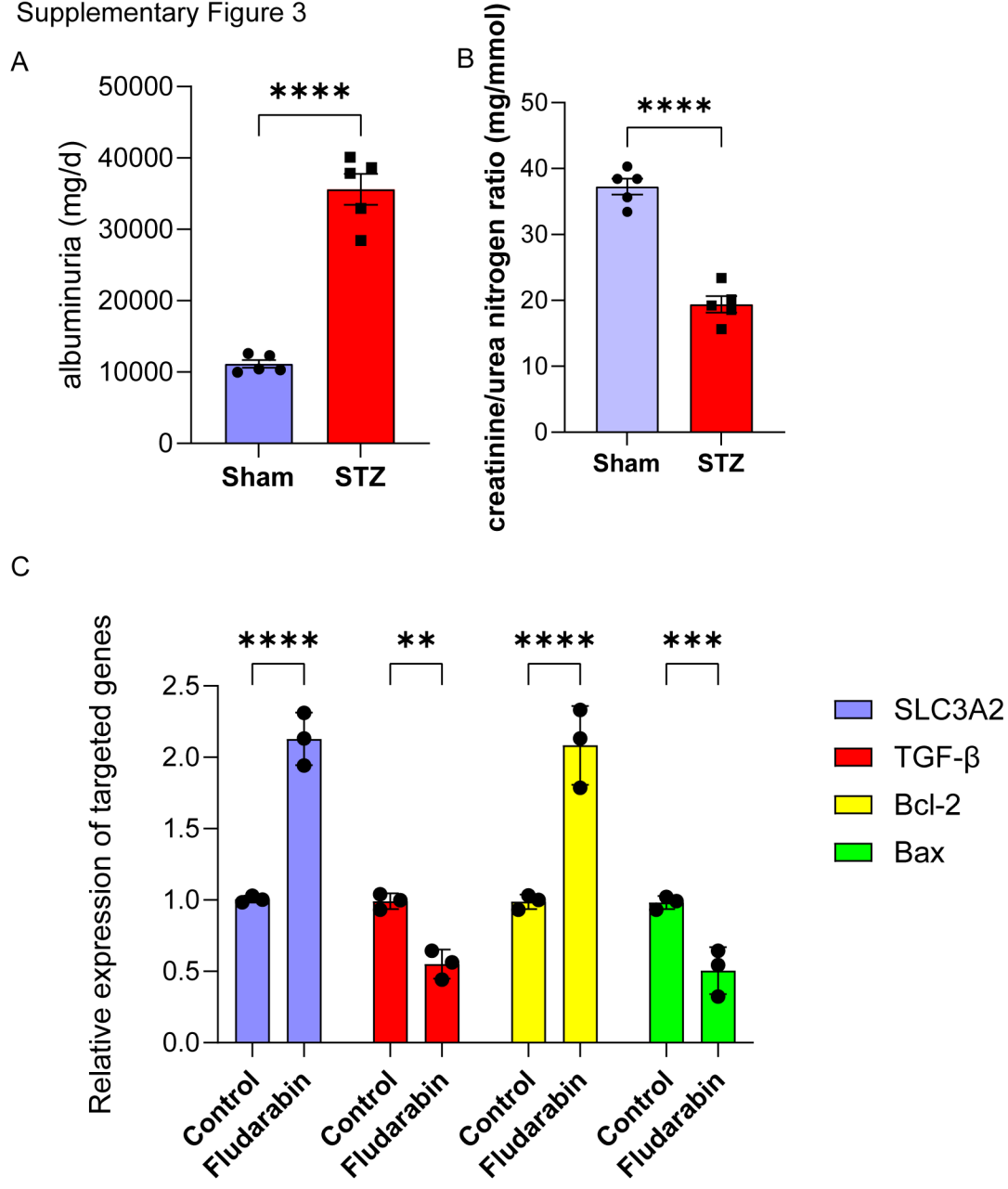

Supplement: Supporting Information 5 — Supporting Figure 3A: Relative expression changes of albuminuria in vivo. Supporting Figure 3B: The relative expression change of creatinine/urea nitrogen ratio (mg/mmol) in vivo. Supporting Figure 3C: The relative expression of SLC3A2 and TGF-β Bcl-2, and Bax in the kidney tissue in vivo between the control group and fludarabine injected group. All data are mean ± SE. ∗p < 0.05, ∗∗p < 0.01, and ∗∗∗p < 0.001. [file 1186113.f5.pdf]
